# Supplementary material for: Safety-oriented and explainable machine learning for KSI crash risk prediction: Evidence from the United Kingdom
Source: PLoS One. 2026 Apr 28;21(4):e0347873. doi: 10.1371/journal.pone.0347873 (PMC13124056; doi:10.1371/journal.pone.0347873)
Supplement: Attachment — Submitted filename: Response to Reviewers.docx [file pone.0347873.s004.docx]

**Detailed response to Editor and Reviewer’s Comments**

**Manuscript No.:** PONE-D-26-00177

Title: Safety-Oriented and Explainable Machine Learning for KSI Crash Risk Prediction: Evidence from the United Kingdom.

**Authors:** Khanh Giang Le

First, I would like to thank the editor for considering my research and the reviewers for your highly constructive comments, which have helped me to enhance the manuscript. I sincerely appreciated all the suggestions made by the editor and the reviewers. I have carefully reviewed these comments and have substantially revised and expanded the manuscript in accordance with the suggestions in order to improve its content and overall quality. The changes made to the manuscript are highlighted using different colors.

======================**Academic** **Editor Comments=================**

**Comment:** After careful consideration, we feel that it has merit but does not fully meet PLOS ONE’s publication criteria as it currently stands. Therefore, we invite you to submit a revised version of the manuscript that addresses the points raised during the review process.

**Answer:** I sincerely thank you and the reviewers for your time and careful evaluation of my manuscript. I greatly appreciate the constructive and insightful comments, which have helped improve the quality of the paper. I have carefully considered all comments and suggestions from both the editor and reviewers, and have thoroughly revised the manuscript accordingly. All changes have been made to address the concerns raised and to ensure that the manuscript meets the journal’s publication standards.

**Comment 1:** Please ensure that your manuscript meets PLOS ONE's style requirements, including those for file naming. The PLOS ONE style templates can be found at

https://journals.plos.org/plosone/s/file?id=wjVg/PLOSOne_formatting_sample_main_body.pdf and https://journals.plos.org/plosone/s/file?id=ba62/PLOSOne_formatting_sample_title_authors_affiliations.pdf

**Answer:** Thank you for this comment. The manuscript has been revised to comply with the PLOS ONE formatting and style requirements, including file naming conventions, as specified in the journal guidelines and templates.

**Comment 2:** Please note that PLOS One has specific guidelines on code sharing for submissions in which author-generated code underpins the findings in the manuscript. In these cases, we expect all author-generated code to be made available without restrictions upon publication of the work. Please review our guidelines at https://journals.plos.org/plosone/s/materials-and-software-sharing#loc-sharing-code and ensure that your code is shared in a way that follows best practice and facilitates reproducibility and reuse.

**Answer:** Thank you for this comment. In accordance with the PLOS ONE guidelines on code sharing, all author-generated code underlying the findings of this study has been made publicly available without restriction. The code has been deposited in a public repository (Zenodo) and can be accessed at: [<https://doi.org/10.5281/zenodo.19135623>]. This ensures full transparency, reproducibility, and reuse of the results.

**Comment 3:** Thank you for uploading your study's underlying data set. Unfortunately, the repository you have noted in your Data Availability statement does not qualify as an acceptable data repository according to PLOS's standards. At this time, please upload the minimal data set necessary to replicate your study's findings to a stable, public repository (such as figshare or Dryad) and provide us with the relevant URLs, DOIs, or accession numbers that may be used to access these data. For a list of recommended repositories and additional information on PLOS standards for data deposition, please see https://journals.plos.org/plosone/s/recommended-repositories.

**Answer:** Thank you for this comment. In response to the journal’s data sharing requirements, the underlying dataset has been uploaded to a stable and publicly accessible repository that complies with PLOS ONE standards. The dataset is available at: [<https://doi.org/10.5281/zenodo.19135778>]. The deposited data include the minimal dataset required to replicate the findings reported in this study. The Data Availability Statement has been updated accordingly to ensure full transparency and reproducibility.

**Comment 4:** We note you have included a table to which you do not refer in the text of your manuscript. Please ensure that you refer to Table 3 in your text; if accepted, production will need this reference to link the reader to the Table.

**Answer:** Thank you for this comment. The manuscript has been revised to include an explicit reference to Table 3 in the main text. This ensures proper linkage between the text and the table for clarity and consistency.

**Comment 5:** If the reviewer comments include a recommendation to cite specific previously published works, please review and evaluate these publications to determine whether they are relevant and should be cited. There is no requirement to cite these works unless the editor has indicated otherwise.

**Answer:** Thank you for this comment. I have carefully reviewed the recommended references and found them to be relevant to the scope of this study. Accordingly, these references have been incorporated into the revised manuscript where appropriate to strengthen the literature context.

**Comment 6:** Please review your reference list to ensure that it is complete and correct. If you have cited papers that have been retracted, please include the rationale for doing so in the manuscript text, or remove these references and replace them with relevant current references. Any changes to the reference list should be mentioned in the rebuttal letter that accompanies your revised manuscript. If you need to cite a retracted article, indicate the article’s retracted status in the References list and also include a citation and full reference for the retraction notice.

**Answer:** Thank you for this comment. The reference list has been carefully reviewed and updated to ensure completeness and accuracy. No retracted articles are included in the revised manuscript. Any necessary corrections and updates to the references have been made accordingly.

==========================**Reviewer #1=======================**

**Comment:** Overall Recommendation: Minor Revision

The manuscript proposes a safety-oriented and explainable machine learning (ML) framework for predicting Killed or Seriously Injured (KSI) crashes using nationwide UK traffic accident data from 2020 to 2024. By framing the problem as a binary classification (KSI vs. slight injury) and systematically addressing class imbalance, decision threshold optimisation, probability calibration, and model interpretability, the study offers a meaningful contribution to the field of traffic safety analytics. However, several issues should be addressed to strengthen before final acceptance.

**Answer:** I sincerely thank the reviewer for the positive evaluation of my study and for recognising the potential contribution of the proposed safety-oriented and explainable machine learning framework to traffic safety analysis. I appreciate the reviewer’s constructive comments and suggestions. All comments have been carefully considered, and the manuscript has been revised accordingly to address the issues raised. All additions and revisions in the revised manuscript have been highlighted in green for clarity**.** Detailed responses to each comment are provided below.

**Comment 1:** Please clarify whether feature selection was performed before modelling.

**Answer:** Thank you very much for your constructive and insightful comments.

I have clarified the feature selection procedure in Section (Data Preprocessing and Feature Engineering) of the revised manuscript. Feature selection was conducted prior to model training using a domain-informed filtering strategy. Specifically, non-informative administrative identifiers, redundant spatial coordinate systems, and post-crash outcome variables were removed to prevent information leakage and ensure that only explanatory variables available before crash occurrence were used in model training. The revised manuscript now explicitly describes these filtering criteria and provides representative examples of the excluded variables.

Changes made in the revised manuscript (Section: Data Preprocessing and Feature Engineering):

…“Feature selection was conducted prior to model training using a domain-informed filtering strategy. This filtering process was designed to eliminate variables that may introduce information leakage and to retain interpretable predictors relevant to traffic safety analysis.

Specifically, variables were systematically evaluated and removed based on three criteria. First, administrative identifiers (e.g., collision_index and collision_ref_no) were excluded because they are non-informative record labels that do not contain meaningful predictive information for crash severity modeling. Second, redundant coordinate systems (e.g., location_easting_osgr and location_northing_osgr) were removed to avoid duplicate spatial representations. Standard geographic coordinates (latitude and longitude) were retained as the primary spatial references, allowing the tree-based model to learn broader spatial patterns and regional safety variations. Finally, post-crash outcome variables (e.g., number_of_casualties and collision_adjusted_severity_serious) were excluded. These post-event variables represent consequences of the crash rather than predictive risk factors, and their inclusion would introduce data leakage into the modeling process.

After this domain-driven filtering, all remaining variables—representing roadway characteristics, environmental conditions, and temporal attributes—were retained without further feature elimination. To further enhance model learning, temporal variables were decomposed into interpretable components (month and hour of occurrence) to capture seasonal and diurnal traffic patterns. Missing values were subsequently addressed using mode imputation for categorical attributes and median imputation for numerical ones [22]. This preprocessing ensures data consistency and improves model robustness.”…

**Comment 2:** Please clarify the hyperparameter settings for the adopted models.

**Answer:** Thank you very much for your constructive and insightful comments. I agree that clearly specifying the hyperparameter settings is important for transparency and reproducibility.

In the revised manuscript, I have added a detailed description of the model configurations in Section (Model Development Using LightGBM). The manuscript has been revised accordingly.

Specifically, for the proposed LightGBM model, the main parameters include a learning rate of 0.03, 1000 boosting iterations, num_leaves = 40, an unrestricted maximum depth (max_depth = −1), and early stopping based on validation loss with 100 rounds.

For comparative evaluation, the baseline models were configured as follows: Logistic Regression with L2 regularization and max_iter = 1000; Random Forest with 100 trees and max_depth = 20; and XGBoost with 1000 boosting rounds, learning_rate = 0.03, max_depth = 6, and tree_method = "hist". These settings ensure a consistent and fair comparison across models.

Changes made in the revised manuscript (Section: Model Development Using LightGBM):

…“To rigorously evaluate the effectiveness of the proposed LightGBM model, several widely used machine learning algorithms, namely Logistic Regression, Random Forest, and XGBoost, were implemented as baseline models for comparative analysis.

To ensure reproducibility and a fair comparison across all algorithms, the hyperparameters were explicitly configured. For the proposed LightGBM model, the main parameters included a learning rate of 0.03, 1000 boosting iterations, and a tree structure controlled by num_leaves = 40 with an unrestricted maximum depth (max_depth = −1).

For the baseline models, configurations were set as follows: Logistic Regression was implemented with L2 regularization and max_iter = 1000; Random Forest used 100 trees with a maximum depth of 20; and XGBoost was trained with 1000 boosting rounds, learning_rate = 0.03, max_depth = 6, and tree_method = "hist".

These configurations follow commonly adopted settings in tabular machine learning tasks, ensuring both model stability and fair comparison across algorithms.”…

**Comment 3:** The reported ROC-AUC value is 0.664. The authors could provide a more apparent justification for why this level is sufficient for safety-critical applications, perhaps by citing similar studies in the literature.

**Answer:** Thank you very much for your constructive and insightful comments. I fully agree that providing contextual justification for the reported ROC–AUC value is important, particularly for safety-critical applications.

In the revised manuscript, I have added a new subsection entitled “Contextual Interpretation of Model Performance” to clarify this point and to position the model performance within the broader crash severity modeling literature. Specifically, I explain that crash severity prediction is inherently challenging due to the stochastic and highly heterogeneous nature of traffic accidents. As discussed by Savolainen et al. (2011), crash outcomes are influenced by numerous interacting factors and substantial unobserved heterogeneity, which naturally limits the achievable predictive accuracy of statistical and machine learning models.

To further contextualize the obtained performance, I also cite recent empirical studies applying machine learning to crash severity prediction. For example, Komol et al. (2021) reported test accuracies ranging from 64.45% to 72.30% when applying Random Forest, SVM, and KNN models to vulnerable road user crash severity classification. Similarly, a recent LightGBM-based fatality prediction study (Rifat et al., 2024) reported a maximum ROC–AUC of approximately 0.72. These findings indicate that predictive performance in the range of AUC ≈ 0.60–0.72 is commonly observed for real-world crash severity modeling problems.

Furthermore, my study adopts a safety-oriented modeling strategy, in which the decision threshold is optimized (τ = 0.35) to improve the detection of severe crashes. As a result, the proposed LightGBM model achieves a Recall of 0.605 for the KSI (Killed or Seriously Injured) class, meaning that more than 60% of severe crashes are correctly identified. In practical traffic safety management, minimizing false negatives (i.e., failing to detect high-risk crashes) is often more critical than marginal improvements in global discrimination metrics.

These explanations and supporting references have been added in (Section: Contextual Interpretation of Model Performance) of the revised manuscript to clarify why the obtained ROC–AUC value is consistent with the expected performance range for crash severity prediction tasks.

Changes made in the revised manuscript (Section: Contextual Interpretation of Model Performance):

“Contextual Interpretation of Model Performance

Crash severity prediction is inherently challenging due to the complex and stochastic nature of road traffic crashes. Unlike many prediction tasks in controlled domains, traffic accidents are influenced by numerous interacting factors, including roadway characteristics, driver behavior, vehicle conditions, environmental factors, and random circumstances occurring at the moment of collision. As highlighted by Savolainen et al. (2011), crash severity outcomes are strongly affected by unobserved heterogeneity and behavioral uncertainty, which significantly limits the achievable predictive accuracy of statistical and machine learning models [26]. Consequently, moderate predictive performance is commonly reported in the crash severity literature.

Empirical evidence from recent machine learning studies supports this observation. For instance, Komol et al. (2021) applied Random Forest, Support Vector Machine, and K-Nearest Neighbor models to analyze crash severity of vulnerable road users using Queensland crash data and reported test accuracies ranging from 64.45% to 72.30%, depending on the road user group considered [31]. Similarly, Rifat et al. (2024) reported that a recent LightGBM-based fatality prediction model achieved a maximum ROC–AUC of approximately 0.72 when comparing multiple machine learning algorithms for traffic accident fatality classification [32]. These findings suggest that predictive performance in the range of AUC ≈ 0.60–0.72 is commonly observed in real-world crash severity modeling problems.

Within this context, the ROC–AUC value obtained in this study (0.664) is consistent with the performance range reported in the literature [31,32]. Rather than being interpreted against a universal benchmark, this result should be understood in relation to the inherent complexity and stochastic nature of crash severity prediction.

Accordingly, the focus of this study is not solely on maximizing global discrimination metrics, but on developing a safety-oriented modeling framework. By optimizing the decision threshold to 0.35, the proposed LightGBM model achieves a Recall of 0.605 for the KSI class, successfully identifying a substantial proportion of severe crashes despite class imbalance.

This perspective highlights that model performance in crash severity prediction should be interpreted in a context-aware manner, where moderate ROC–AUC values can still provide meaningful and actionable insights when aligned with safety-critical objectives and decision-making requirements.”…

**Comment 4:** There is no direct comparison with baseline models (e.g., logistic regression, random forest, XGBoost).

**Answer:** Thank you very much for this valuable suggestion. I agree that comparing the proposed model with commonly used baseline algorithms is important to contextualize the performance of the proposed framework.

In the revised manuscript, I have implemented three widely used baseline models including Logistic Regression, Random Forest, and XGBoost, and conducted a comprehensive comparative evaluation with the proposed LightGBM model. The implementation details and parameter configurations for these baselines are now described in (Section: **Model development using LightGBM**). The results are now presented in **Section (Comparative Performance with Baseline Models)** and summarized in **Table 3**, where multiple evaluation metrics are reported, including ROC–AUC, PR–AUC, Brier score, Precision (KSI), Recall (KSI), and F1-score.

The comparative analysis demonstrates that the proposed LightGBM model achieves the highest ROC–AUC and Precision for KSI detection while also providing the lowest Brier score, indicating strong discriminative capability and reliable probabilistic predictions. These additions provide a clearer empirical justification for selecting LightGBM as the final model.

The manuscript has been revised accordingly.

Changes made in the revised manuscript (Section: **Comparative Performance with Baseline Models**):

**Comparative Performance with Baseline Models**

…“Table 5 presents the comparative performance of the evaluated machine learning models at the safety-optimized decision threshold (τ* = 0.35). The comparison includes the proposed LightGBM model and three widely used baseline algorithms: Logistic Regression, Random Forest, and XGBoost.

Table 5. Comparative performance of machine learning models for KSI crash prediction at the safety-optimized decision threshold (τ* = 0.35).

| Model | ROC–AUC | PR–AUC | Brier Score ↓ | Precision (KSI) | Recall (KSI) | F1-score |
| --- | --- | --- | --- | --- | --- | --- |
| Logistic Regression | 0.636 | 0.334 | 0.227 | 0.282 | 0.808 | 0.418 |
| Random Forest | 0.660 | 0.345 | 0.201 | 0.311 | 0.704 | 0.432 |
| XGBoost | 0.663 | 0.350 | 0.192 | 0.325 | 0.621 | 0.427 |
| LightGBM (Proposed) | 0.664 | 0.349 | 0.190 | 0.328 | 0.605 | 0.425 |

As commonly observed in crash severity modeling, no single algorithm dominates all evaluation metrics due to the inherent trade-off between precision and recall. For example, Logistic Regression achieved the highest Recall (0.808), but this occurred at the expense of substantially lower Precision (0.282), implying a high rate of false alarms.

Among the evaluated models, LightGBM achieved the highest ROC–AUC (0.664), indicating the highest overall discriminative performance among the evaluated models. In addition, LightGBM achieved the highest Precision for KSI detection (0.328) while maintaining competitive Recall and F1-score values. The PR–AUC values are also comparable across the evaluated models, with XGBoost and LightGBM achieving the highest scores (approximately 0.35), indicating robust performance under class imbalance.

Importantly, LightGBM also produced the lowest Brier score (0.190), indicating superior probabilistic reliability compared with Random Forest (0.201) and XGBoost (0.192). These results suggest that the LightGBM model provides a balanced combination of discrimination and probabilistic reliability, making it particularly suitable for safety-oriented crash risk prediction.

The comparative analysis confirms that the proposed LightGBM model provides a strong balance between discriminative performance and probabilistic reliability at the safety-optimized threshold. Building on this foundation, the next section further investigates the model's probabilistic reliability through calibration analysis, which is essential for risk-based traffic safety management.”

**Comment 5:** The finding that “good road surface conditions” and “fine weather” are associated with higher KSI risk is intriguing but requires deeper scrutiny. The authors could find support from previous studies.

**Answer:** Thank you very much for your constructive and insightful comments. I agree that these counterintuitive findings warrant further explanation supported by previous studies.

To address this concern, I have expanded the discussion in Section (Integrated Discussion and Practical Implications) to interpret these patterns through the lens of behavioural adaptation and risk compensation mechanisms. Previous studies suggest that favourable driving conditions may create a false sense of safety, which can encourage higher travel speeds and reduced vigilance, thereby increasing the likelihood of severe crash outcomes when collisions occur. In contrast, adverse conditions typically prompt drivers to reduce speeds, which may mitigate crash severity despite the potentially higher crash occurrence reported in prior studies.

Specifically, I have revised and added the following discussion to the manuscript to clarify this point:

Changes made in the revised manuscript (Section: Integrated Discussion and Practical Implications):

…“ Meanwhile, several counterintuitive patterns—such as higher KSI risk under good weather or dry road conditions—warrant further scrutiny. These patterns have been widely discussed in the traffic safety literature and are commonly interpreted through behavioral adaptation and risk compensation mechanisms [35,36]. According to risk compensation theory, drivers may adjust their behavior to maintain an acceptable level of perceived risk. For instance, under adverse weather or slippery road conditions, drivers typically perceive higher risk and may compensate by driving more cautiously, often reducing travel speeds. As a result, although crash frequency may increase under such unfavorable conditions, previous studies have reported that crash severity may be mitigated by lower impact speeds [37]. Conversely, favorable environmental conditions can create a false sense of safety, encouraging higher travel speeds and reduced vigilance. When collisions occur under these conditions, the higher kinetic energy associated with greater travel speeds substantially increases the likelihood of severe outcomes, including KSI crashes. Importantly, these patterns reflect conditional risk relationships learned from observational data rather than direct causal effects, highlighting the importance of careful interpretation when translating model outputs into safety policies.”…

**Comment 6:** The authors could emphasise the linkage between safety-oriented thresholds and real-world intervention strategies. Please guide the transport authorities on operationalising threshold-based alerts.

**Answer:** Thank you very much for your constructive and insightful comments. I appreciate this suggestion, as linking predictive metrics with actionable safety interventions is an important objective of this research.

I agree that providing clearer guidance on how safety-oriented thresholds can be operationalized can enhance the practical relevance of the study. In response, I have expanded Section (Integrated Discussion and Practical Implications) to clarify how the proposed thresholds can support real-world traffic management and decision-making processes.

Specifically, the revised manuscript now illustrates potential deployment scenarios in which predicted crash severity probabilities can be integrated into real-time Traffic Management Center (TMC) systems to trigger threshold-based alerts and support proactive interventions.

Changes made in the revised manuscript (Section: Integrated Discussion and Practical Implications):

…“ To operationalize these safety-oriented thresholds, transportation authorities could integrate the proposed framework into real-time traffic management systems. In practice, contextual crash information (e.g., location, roadway characteristics, weather conditions, and traffic environment) can be rapidly processed by the model to estimate the probability of a severe crash outcome. When the predicted probability exceeds a predefined high-risk threshold, the system may automatically trigger an alert within Traffic Management Centers (TMCs) or emergency coordination platforms.

Such threshold-based alerts can support proactive decision-making, including issuing driver warnings through variable message signs, prioritizing incident management resources, or coordinating emergency response services. By translating model predictions into operational alert levels, the proposed framework provides a practical mechanism for transforming predictive analytics into actionable traffic safety interventions.”…

**Comment 7:** The data covers 2020–2024, which includes the COVID-19 pandemic period. The authors should briefly discuss whether and how pandemic-induced changes in traffic patterns might affect model training.

**Answer:** Thank you very much for your constructive and insightful comments. I agree that the study period (2020–2024) partially overlaps with the COVID-19 pandemic, during which traffic demand and travel behavior experienced temporary disruptions in many regions.

In response to the reviewer’s suggestion, I have added a brief discussion in Section (Integrated Discussion and Practical Implications) to acknowledge the potential influence of pandemic-related mobility changes on crash patterns and model training.

The revised manuscript now clarifies that reduced traffic volumes and altered travel behavior during certain pandemic phases may have influenced the distribution of crash characteristics used in the dataset. At the same time, the multi-year dataset includes both pandemic and post-pandemic traffic conditions, which helps mitigate the influence of short-term anomalies. The added discussion also highlights that future research could further investigate these effects through more detailed temporal segmentation of crash data.

Changes made in the revised manuscript (Section: Integrated Discussion and Practical Implications):

The added text is reproduced below for clarity:

…“Finally, it is important to acknowledge that the study period (2020–2024) partially overlaps with the COVID-19 pandemic, during which traffic demand and travel behavior experienced temporary disruptions in many regions. Reduced traffic volumes, changes in travel purposes, and altered mobility patterns during certain pandemic phases may have influenced the distribution of crash characteristics used for model training. However, the dataset spans multiple years and includes both pandemic and post-pandemic traffic conditions, which helps mitigate the influence of short-term anomalies. Consequently, the model is more likely to capture broader crash severity patterns rather than behaviors specific to pandemic-related travel conditions. Future research could further examine the potential impact of pandemic-induced mobility changes through more detailed temporal segmentation of crash data.”…

**--- This is the end of the detailed response for all suggestions from reviewer #1 ---**

========================== **Reviewer #2=======================**

**Answer:** I sincerely thank the reviewer for constructive comments and suggestions. All comments have been carefully considered, and the manuscript has been revised accordingly to address the issues raised. All additions and revisions in the revised manuscript have been highlighted in Turquoise for clarity**.** Detailed responses to each comment are provided below.

**Comment 1:** Literature review could be strengthened. The related work section would benefit from a clearer synthesis of prior studies on imbalance handling, threshold selection, and probability calibration in crash severity modeling such as

<https://doi.org/10.1016/j.ijtst.2024.11.009> and

<https://doi.org/10.1016/j.aap.2025.108277>

Explicitly positioning this study relative to those strands would better highlight its contribution.

**Answer:** Thank you very much for this constructive suggestion. I agree that strengthening the literature review and more clearly positioning the proposed framework within existing crash severity research improves the clarity and contribution of the study.

Following the reviewer’s recommendation, I have expanded the Introduction and Related Work section to incorporate recent crash severity studies that emphasize temporal heterogeneity and instability in crash risk factors. In particular, I have added discussion and citations to recent research employing random-parameter logit models that demonstrate how key risk factors (e.g., lighting conditions, speed limits, and roadway characteristics) may exhibit temporal instability and seasonal variation.

These additions help clarify that crash severity mechanisms are inherently heterogeneous and dynamic, which presents significant challenges for reliable prediction and motivates the use of data-driven machine learning approaches capable of capturing complex nonlinear relationships.

Changes made in the revised manuscript (Section: Introduction and related work):

…“ Recent studies employing random-parameter logit models have made important advances by capturing temporal instability and seasonal variation in key risk factors, such as lighting conditions, speed limits, and roadway characteristics [6,7]. These findings highlight the inherently dynamic and heterogeneous nature of crash severity, posing challenges for reliable prediction and risk-based safety assessment.’’…

**Comment 2:** Decision threshold optimization needs clearer justification. The choice of optimizing Recall(KSI) is reasonable, but the practical constraint on precision is only discussed qualitatively. A brief quantitative justification or sensitivity check would strengthen the methodological rigor.

**Response:**
We sincerely thank the reviewer for this insightful and constructive comment. We fully agree that the practical constraint on precision should be supported by a clear quantitative justification rather than a qualitative discussion.

In the revised manuscript, we have strengthened this aspect by introducing a quantitative, data-driven threshold optimization framework and an explicit sensitivity analysis.

First, threshold selection is now formulated as a constrained optimization problem using a penalty-based Operational Utility Score (OUS), which maximizes Recall(KSI) while penalizing solutions that violate a minimum precision requirement (P_target = 0.33). This threshold corresponds to an approximate true-to-false alarm ratio of 1:2, ensuring that excessive false alarms are explicitly controlled.

Second, we conducted a systematic sensitivity analysis across candidate thresholds (τ = 0.20–0.50). The results, now presented in **Table 2**, quantitatively demonstrate that:

- Lower thresholds (τ ≤ 0.30) achieve high recall but incur substantial penalties due to insufficient precision, indicating excessive false alarms;
- Higher thresholds (τ ≥ 0.40) satisfy the precision constraint but significantly reduce recall;
- The selected threshold (τ* = 0.35) achieves the **maximum Operational Utility Score (OUS = 0.586)**, representing the best trade-off between detection capability and false-alarm control.

Importantly, the selected threshold slightly relaxes the precision target (0.328 vs. 0.33) with only a negligible penalty, while preserving a high recall (0.605). This demonstrates that the final threshold is not determined heuristically, but is quantitatively justified based on sensitivity analysis under explicit operational constraints.

The corresponding revisions have been incorporated as follows:

- **(Section: Decision threshold optimization):** Introduction of the OUS-based constrained optimization framework;

…“ However, threshold selection inherently involves a trade-off between sensitivity (Recall) and predictability (Precision). As emphasized by Xu et al. (2016), lowering the threshold improves the detection of hazardous conditions but inevitably increases the false alarm rate, reflecting the fundamental trade-off between sensitivity and predictability [29]. From a human factors perspective, excessive false alarms can erode user trust and lead to alarm fatigue, thereby reducing operational effectiveness [30].

To avoid subjective decision-making, threshold selection was formulated as a constrained optimization problem using a penalty-based approach. Predicted KSI probabilities were evaluated across a range of candidate thresholds τ ∈ [0,1]. An Operational Utility Score (OUS) is defined to maximize Recall while applying a penalty coefficient (λ = 10) when Precision falls below an operational target ($P_{\mathrm{target}}$ = 0.33), corresponding to a true-to-false alarm ratio of approximately 1:2. This formulation penalizes excessive false alarms without rigidly discarding near-optimal solutions.

The objective function is defined as follows:

$OUS(\tau)= Recall(\tau)- \lambda. max(0, P_{\mathrm{target}}- Precision(\tau))$	(2)

The optimal threshold is therefore selected as:

τ* = argmax_τ OUS(τ)	(3)

This formulation explicitly reflects the predictability–sensitivity trade-off described in Xu et al. (2016), where improving sensitivity inevitably increases the false alarm rate [29]. The proposed approach replaces heuristic threshold selection with a data-driven optimization strategy, ensuring a balanced trade-off between detection performance and false-alarm control.”…

- **(Section: Decision threshold optimization and confusion matrix analysis):** Addition of a quantitative sensitivity analysis and Table 2:

…“ To provide a quantitative justification for the threshold selection, a sensitivity analysis was conducted across candidate thresholds ranging from 0.20 to 0.50. The results are summarized in Table 2.

Table 2. Quantitative threshold optimization based on sensitivity–predictability trade-off.

| Threshold (τ) | Precision | Recall | OUS |
| --- | --- | --- | --- |
| 0.20 | 0.286 | 0.867 | 0.430 |
| 0.25 | 0.302 | 0.790 | 0.511 |
| 0.30 | 0.315 | 0.699 | 0.554 |
| 0.35 | 0.328 | 0.605 | 0.586 |
| 0.40 | 0.342 | 0.514 | 0.514 |
| 0.50 | 0.366 | 0.349 | 0.349 |

As shown in Table 2, lower thresholds (τ ≤ 0.30) achieve high recall (0.699–0.867) but incur heavy penalties due to low precision, resulting in reduced OUS values and indicating excessive false alarm rates. In contrast, higher thresholds (τ ≥ 0.40) avoid penalties but exhibit a significant reduction in recall (down to 0.349–0.514), limiting the model’s ability to detect severe crashes.

The threshold τ* = 0.35 emerges as the optimal operating point. Its precision (0.328) is slightly below the target (0.33), incurring only a minimal penalty. In exchange, the model retains a relatively high recall of 0.605, leading to the maximum OUS (0.586). This result indicates that τ* = 0.35 provides an effective data-driven trade-off between detection capability and false-alarm control for practical deployment.”…

I believe this addition directly addresses the reviewer’s concern and significantly strengthens the methodological rigor of the study.

**Comment 3:** Justification for SMOTE should be clearer. While SMOTE is adopted as the final imbalance-handling strategy, comparative results against alternative approaches such as class-weighted learning are not shown. A concise comparison would improve transparency.

**Answer:** I sincerely thank the reviewer for this valuable suggestion. To improve transparency, I have added a comparative analysis of imbalance-handling strategies, including SMOTE and class-weighted learning, in the revised manuscript (see Table 4).

The comparison was conducted under the same decision threshold (τ* = 0.35) to ensure a fair and operationally consistent evaluation. The results show that class-weighted learning achieves a substantially higher Recall (0.904), but at the cost of significantly reduced Precision (0.284), leading to an excessive false alarm rate.

In contrast, the SMOTE-based model provides a more balanced performance, achieving higher Precision (0.328) while maintaining competitive Recall (0.605). In addition, SMOTE yields a lower Brier score (0.190 vs. 0.221), indicating more reliable probability estimates. When evaluated using the proposed Operational Utility Score (OUS), which explicitly incorporates both recall maximization and precision constraints, SMOTE achieves the highest overall utility (0.586), outperforming class-weighted learning (0.445).

These results demonstrate that while class-weighted learning prioritizes sensitivity, it does not satisfy practical operational requirements due to excessive false alarms. SMOTE, on the other hand, provides a more effective trade-off between detection capability and false-alarm control, making it more suitable for safety-critical traffic applications.

Accordingly, SMOTE was selected as the primary imbalance-handling strategy in the final model. The corresponding explanation has been added to (Sections: Comparative performance with baseline models) and (Section: Comparative performance with baseline models) of the revised manuscript.

- Sections: Comparative performance with baseline models

…“ In addition to SMOTE, class-weighted learning was considered as an alternative imbalance-handling strategy. Class-weighted learning operates at the algorithm level by modifying the loss function to penalize misclassification of the minority class, whereas SMOTE modifies the data distribution through synthetic sample generation.

In this study, SMOTE is adopted as the primary imbalance-handling strategy in the final model. Class-weighted learning is implemented as a comparative approach only, and is not used in the final model configuration. A quantitative comparison between these strategies is presented in the Results section to evaluate their impact on predictive performance and operational effectiveness. The final selection of SMOTE is therefore based on empirical evidence rather than methodological preference.”…

- Sections: Comparative performance with baseline models

“Before comparing different machine learning algorithms, it is important to clarify the impact of imbalance-handling strategies on model performance. To improve transparency, a comparative analysis between SMOTE and class-weighted learning was conducted under the same decision threshold (τ* = 0.35). The results are presented in Table 4.

Table 4. Comparison of imbalance-handling strategies.

| Imbalance Strategy | ROC–AUC | Brier Score ↓ | Precision (KSI) | Recall (KSI) | OUS |
| --- | --- | --- | --- | --- | --- |
| Class-Weighted Learning | 0.692 | 0.221 | 0.284 | 0.904 | 0.445 |
| SMOTE (Proposed) | 0.664 | 0.190 | 0.328 | 0.605 | 0.586 |

As shown, class-weighted learning achieves a substantially higher Recall (0.904), indicating strong sensitivity to KSI crashes. However, this comes at the expense of a significantly reduced Precision (0.284), reflecting an excessive false alarm rate. In safety-critical applications, such behavior may lead to alarm fatigue and reduced operational trust.

In contrast, the SMOTE-based model provides a more balanced performance, achieving a higher Precision (0.328) while maintaining a reasonable Recall (0.605). In addition, SMOTE yields a substantially lower Brier score (0.190 vs. 0.221), indicating more reliable probability estimates for risk-based decision-making. Importantly, when evaluated using the proposed Operational Utility Score (OUS), which explicitly incorporates both recall and precision constraints, SMOTE achieves the highest utility (0.586), outperforming class-weighted learning (0.445).

These results indicate that although class-weighted learning maximizes sensitivity, it does so at the cost of operational feasibility. SMOTE, on the other hand, provides a more effective trade-off between detection capability and false-alarm control, making it more suitable for real-world deployment in traffic safety systems.”…

**Comment 4:** ROC–AUC interpretation should be more cautious. The discussion of the achieved AUC would benefit from clearer framing as being consistent with prior crash severity studies, rather than implying a general performance benchmark.

**Answer:** I thank the reviewer for this insightful comment. I agree that ROC–AUC values in crash severity prediction should be interpreted with caution and within the context of domain-specific complexity.

In the revised manuscript, we have removed statements implying a universal performance benchmark (e.g., fixed AUC thresholds) and instead framed the achieved ROC–AUC in relation to prior studies. Specifically, I clarified that the obtained ROC–AUC (0.664) is consistent with previously reported ranges (approximately 0.60–0.72) in crash severity prediction literature [30,31], where predictive performance is inherently constrained by unobserved behavioral factors and stochastic variability.

These revisions have been incorporated in Section (ROC–AUC Analysis) and further reinforced in Section (Contextual Interpretation of Model Performance) to ensure a more cautious and context-aware interpretation of model performance.

- Section : ROC–AUC Analysis

…“ Previous studies have reported ROC–AUC values in the range of approximately 0.60–0.72 for crash severity prediction under real-world conditions [31,32]. Within this context, the performance achieved in this study falls within the expected range and demonstrates that the proposed model captures statistically meaningful risk patterns despite the inherent uncertainty of crash outcomes.”…

- Section : Contextual Interpretation of Model Performance

…“ Within this context, the ROC–AUC value obtained in this study (0.664) is consistent with the performance range reported in the literature [31,32]. Rather than being interpreted against a universal benchmark, this result should be understood in relation to the inherent complexity and stochastic nature of crash severity prediction.

Accordingly, the focus of this study is not solely on maximizing global discrimination metrics, but on developing a safety-oriented modeling framework. By optimizing the decision threshold to 0.35, the proposed LightGBM model achieves a Recall of 0.605 for the KSI class, successfully identifying a substantial proportion of severe crashes despite class imbalance.

This perspective highlights that model performance in crash severity prediction should be interpreted in a context-aware manner, where moderate ROC–AUC values can still provide meaningful and actionable insights when aligned with safety-critical objectives and decision-making requirements.”…

**Comment 5:** SHAP interpretations should emphasize non-causality. Although the SHAP analysis is informative, some interpretations may be influenced by reporting or behavioral factors. A clearer distinction between associative patterns and actionable mechanisms would improve clarity.

**Answer:** I thank the reviewer for this important comment. I agree that SHAP-based interpretations should clearly distinguish between associative patterns and causal mechanisms.

In the revised manuscript, I have strengthened the interpretation of SHAP results by explicitly emphasizing that SHAP values capture statistical associations rather than causal relationships. I have also clarified that some observed patterns may be influenced by reporting practices or behavioral responses, particularly for variables such as police attendance and weather conditions.

Furthermore, I have added a dedicated paragraph in (Section: Model explainability using SHAP) to distinguish between associative signals and actionable mechanisms, highlighting that the SHAP results are intended for risk identification and prioritization rather than causal inference. These revisions improve the clarity and methodological rigor of the model interpretation.

Changes made in the revised manuscript (Section: Model explainability using SHAP):

…“ Table 6. Interpretation of SHAP patterns for selected explanatory variables (non-causal, risk-oriented interpretation).

| Variable | STATS19 coding | SHAP pattern | Interpretation |
| --- | --- | --- | --- |
| did_police_officer_  attend_scene_of  _accident | No = 1; Yes = 2 | Police attendance → SHAP < 0 | Crashes without recorded police attendance are more frequently associated with higher predicted KSI risk, which may reflect reporting or post-event factors rather than causal effects. |
| number_of_vehicles | 1, 2, 3, … | Higher values → SHAP < 0 | Single-vehicle crashes are more frequently associated with higher predicted KSI risk compared with multi-vehicle crashes. |
| road_type | Roundabout=1→One way street→Dual carriageway→Single carriageway→Slip road→One way street/Slip road=7 | Certain types (Slip road, One-way street/Slip road) → SHAP < 0 | Certain road types are associated with lower predicted KSI risk, reflecting differences in geometric design and traffic conditions. |
| road_surface_conditions | Dry=1 → Wet=2; Snow=3; Ice→Flood→Oil→Mud=7 | Better conditions → SHAP > 0 | Better road surface conditions are associated with higher predicted KSI risk, which may reflect higher travel speeds under favorable conditions. |
| speed_limit | 20 → 70 mph | Higher values→ SHAP > 0 | Higher speed limits are strongly associated with increased predicted KSI risk. |
| junction_detail_historic | Roundabout=1; T junction→ Slip road→Crossroads→> 4 arms=7 | More complex junctions → SHAP < 0 | More complex junction layouts are associated with lower predicted KSI risk, potentially reflecting reduced speeds and increased driver caution. |
| first_road_class / second_road_class | 1: Motorway → 2: A(M) → 3: A → 4: B → 5: C → 6: Unclassified | Lower-class roads → SHAP > 0 | Lower-class roads are associated with higher predicted KSI risk, possibly due to roadway design and protection differences. |
| light_conditions | Daylight=1 → Darkness=7 | Darkness → SHAP > 0 | Darkness is associated with higher predicted KSI risk, reflecting reduced visibility conditions. |
| weather_conditions | Fine=1→Rain→Snow→Snowing + high winds →Fog=7 | Higher values → SHAP < 0 | Adverse weather conditions are associated with lower predicted KSI risk, which may reflect behavioral adaptation such as reduced speed. |
| urban_or_rural_area | Urban = 1; Rural = 2 | Urban → SHAP > 0 | Urban areas are associated with higher predicted KSI risk in the observed data. |

It is important to explicitly distinguish between statistical associations identified by the model and actionable safety mechanisms. SHAP values capture patterns present in the observed data, which may be influenced by reporting practices, exposure differences, or behavioral adaptations, rather than direct causal effects.

For example, variables such as police attendance or weather conditions may reflect post-event reporting processes or behavioral responses (e.g., reduced speed under adverse conditions), rather than intrinsic risk factors. Therefore, the observed SHAP patterns should be interpreted as associative signals that help identify high-risk conditions, rather than as direct evidence of causal relationships.

From a practical perspective, these insights are most appropriately used for risk screening and prioritization, while causal interpretation and policy intervention should be supported by domain knowledge and complementary analytical approaches.

Despite its advantages in enhancing model transparency, SHAP-based interpretation has several inherent limitations that should be acknowledged. First, SHAP values describe conditional associations learned from observational data rather than causal relationships, and therefore should not be interpreted as evidence of direct causality. Second, some observed patterns may be influenced by reporting biases or behavioral adaptations rather than underlying risk mechanisms. For example, variables related to police attendance or weather conditions may reflect differences in reporting practices or driver responses rather than intrinsic crash severity determinants. Accordingly, the SHAP results in this study are intended to support risk interpretation and prioritization, while causal inference and policy design should rely on complementary domain-specific analyses.”

**--- This is the end of the detailed response for all comments from reviewer #2 ---**

**Finally, I highly appreciate for the chance the editor and two reviewers have provided me to improve the manuscript, and your valuable feedback has significantly enhanced its quality. I trust that this revised version meets your expectations. Should you have any additional concerns or matters, please inform me.**

**Thank you very much for helping me to improve the manuscript and I hope that you are satisfied with my answers.**
